# Ultrasonographic Characterizations of Radial Artery for Transradial Approach in the Saudi Population: A Pilot Study

**DOI:** 10.7759/cureus.65532

**Published:** 2024-07-27

**Authors:** Bassem Y Sheikh, Mohamed S Abd Elziz, Mohammed K Almuzaini, Khaled A Alhejaili, Eyad K Alharbi, Aseel M Andijany, Feras H Alharbi, Hussam A Alahmadi

**Affiliations:** 1 Neurosurgery, King Salman bin Abdulaziz Medical City, Madinah, SAU; 2 Radiology, Andalusia Hospital, Jeddah, SAU; 3 College of Medicine, Taibah University, Madinah, SAU

**Keywords:** ultrasonography, arterial diameter, transradial approach, radial characterization, radial artery

## Abstract

Background

The radial artery originates from the brachial artery at the level of the neck of the radius in the cubital fossa. It has multiple branches all over its course, and it is an important artery for multiple procedures across several fields.

Objectives

The objective of this article is to assess the size and characteristics of the radial artery in the Saudi population for the transradial approach. It aims to compare the diameters of the distal and proximal radial arteries using ultrasonography and determine the clinical significance of the findings in selecting an appropriate catheter size among different demographic groups. Additionally, the study aims to contrast the obtained results with international standards to provide a comprehensive analysis of the radial artery characteristics in the Saudi population.

Methods

A pilot study was conducted at a Medina region hospital in Madinah from December 2022 to July 2023. An ultrasonographic assessment of the right radial artery was performed on a sample of 45 volunteers.

Results

Our results showed a significantly larger mean diameter of the right ulnar artery in males compared to females. No other significant differences were observed in the characteristics of the right proximal radial artery (PRA), distal radial artery (DRA), or ulnar artery between genders. Significant differences in arterial characteristics were observed across different body mass index (BMI) categories for several parameters. Depth measurements in the right PRA displayed notable differences across age groups, and the ulnar artery showed significant variability among age categories. No statistically significant differences were found in arterial characteristics across smoking categories.

Conclusion

Our study on Saudi Arabian radial artery ultrasonography reveals potential clinical correlations, highlighting the influence of age and BMI on arterial characteristics. Further research is needed to confirm these findings and explore demographic determinants.

## Introduction

The radial artery originates from the brachial artery at the level of the neck of the radius in the cubital fossa. It has multiple branches along its course, including the recurrent radial artery, the palmar carpal branch, the superficial palmar branch, the deep palmar branch, the arteria princeps pollicis, and the arteria radialis indicis. The radial artery initially descends, accompanied by a pair of radial veins. Then, it continues near the superficial branch of the radial nerve. After that, it runs between the pronator teres medially and the brachioradialis laterally and then between the flexor carpi radialis and brachioradialis to reach the distal end of the radius anteriorly, where the radial pulse is felt against the bone. Next, it curves over the radial aspect of the wrist joint, passing under the abductor pollicis longus tendon and reaching the anatomical snuffbox. At this point, the distal radial pulse can be palpated over the scaphoid and trapezium. In the hand, it enters the palm and joins with the deep branch of the ulnar artery, forming the deep palmar arch [1].

The radial artery is an important site for various procedures across several fields. This site is a feasible option for neuroendovascular interventions [2]. Likewise, it can be safely used for acute and non-acute coronary artery interventions [3]. Additionally, it can be used for renal artery interventions as a possible alternative to other approaches [4]. The carotid artery and vertebral artery stenting can be effectively performed using the transradial approach (TRA) [5, 6]. Moreover, it is a possible option in arteriovenous fistuloplasty [3].

In neuroendovascular interventions, the transfemoral approach (TFA) has been the standard for years in diagnostic and therapeutic procedures. TFA is the classical approach due to the limitless number of punctures, the ease of access, and the familiarity among most interventionists. The TRA, which is most commonly used in cardiac interventions, has gained popularity as an alternative to TFA due to its many advantages. The key benefits of this catheterization technique are quick recovery, allowing for early ambulation, fewer postoperative side effects, lower hospital costs, and a lower risk of scarring. However, it requires experience [7].

The ultrasound facilitates the identification of anatomical landmarks and ensures precise vessel access. Radial artery cannulation can be particularly challenging in situations involving small-caliber arteries or anatomical distortion. Recently, the use of US has emerged as a recommended and effective tool for guiding vascular punctures, enhancing the initial success rate on the first attempt and reducing complications in adults compared to the traditional palpation method [8].

Studies show that coronary angiography and angioplasty performed via the radial artery have fewer complications compared to femoral or brachial access [9]. The TRA is associated with lower mortality rates, bleeding problems, major adverse cardiovascular events, and vascular complications in acute coronary syndromes [10]. Bleeding is a problem with femoral access, which can be severe enough in some cases to warrant transfusion. In patients with a bleeding tendency, the TRA reduces the risk of hemorrhage, lowers vascular complications, and reduces pseudoaneurysm compared to the TFA [7].

Catheterization via the radial artery is usually safe. However, thrombosis, infections, and mechanical complications occur in 1% of all patients. With every additional attempt, the probability of complications increases, as does the difficulty of the procedure. Vasospasm and hematoma formation can also complicate and contribute to difficult cannulation. Hypotension, edema, atherosclerosis, and arterial scarring are all risk factors for unsuccessful TRA [11].

Lack of experience with the relatively new TRA is a major obstacle to adopting this technique for neuroendovascular procedures. Another obstacle is the lack of TRA neurointerventional catheter systems on the market, which makes catheter choice difficult for inexperienced practitioners. The senior author introduced TRA in the Kingdom of Saudi Arabia (KSA) in mid-2016, utilizing it for both diagnostic and therapeutic procedures [12].

A Korean study published in 2021 measured the size of the distal radial artery (DRA) in Korean participants. They found that the average diameter of the DRA for men was 2.45, and for women was 2.16 [13]. Another study by Anastasia et al., with a mean age of 64 years, found that the mean size of the left radial artery was 2.55 mm and 2.34 mm at the snuff box (p-value <0.001) [14]. To date, there are no studies illustrating the average size of the radial artery in the Saudi population. We aim to assess the feasibility of such a study in Saudi Arabia.

## Materials and methods

A pilot study (STU-22-21) was conducted in the Madinah region from December 2022 to July 2023. An ultrasonographic assessment of the right radial artery was performed on a sample of 45 volunteers [15]. The diameters of the proximal radial artery (PRA) and distal radial artery (DRA), the radial wall, and the depth of the radial artery from the skin were assessed using an ultrasound probe. The results of the Doppler ultrasound-guided Allen test were recorded to assess the blood flow in the palmar arches. The Allen test was performed following the WHO guidelines [16].

Each volunteer was instructed to lie down in a quiet room, where the measurement of the distal radial artery (DRA) was performed in the snuffbox over the scaphoid and trapezium bones, while the measurement of the proximal radial artery (PRA) was conducted 2 cm proximal to the distal wrist crease, laterally to the flexor carpi radialis tendon. The blood flow pattern observed in the Doppler ultrasound-guided Allen test was categorized into four groups: no flow, decreased flow, reversed flow, and increased flow.

The inclusion criteria consisted of Saudi nationals over 18 years old and individuals without a history of transradial catheterization or a diagnosis of peripheral arterial disease. In addition, Saudi volunteers who met the baseline demographic variables of age, gender, smoking status, diabetes, and hypertension were included. Exclusion criteria encompassed individuals younger than 18 years, those with a history of transradial catheterization, those diagnosed with peripheral vascular disease, or non-Saudi nationality.

Statistical analysis

Statistical analysis was carried out using RStudio version 4.3.0 (R Foundation, Vienna, Austria). Frequencies and percentages were used to present categorical variables, while mean ± standard deviation (SD) was used to express continuous variables. Comparisons between males and females were performed using Fisher's exact test or Pearson's Chi-squared test for categorical variables and the Wilcoxon rank sum test or Wilcoxon rank sum exact test for continuous variables with two or three more categories, respectively. The correlation between the diameters of the PRA and DRA was assessed using Spearman correlation analysis. Statistical significance was defined at p<0.05.

Ethical considerations

This study has been submitted for revision to the research ethics committee of Taibah University. Ethical approval has since been provided. The study will abide by the ethical rules and regulations regarding confidentiality and privacy of information, in addition to obtaining appropriate consent from each participant.

## Results

Demographic and clinical characteristics

In the study cohort (N=45), the mean ± SD age was 29.42 years ± 10.52, and the mean ± SD body mass index (BMI) was 27.57 kg/m2 ± 6.50. Females were significantly older than males (32.39 ± 10.43 vs. 27.44 ± 10.30, p=0.044), while males were significantly taller than females (170.20 ± 5.39 vs. 159.94 ± 6.96, p<0.001). The majority of participants exhibited right-handed dominance (93.3%). Among the participants, 31.1% reported a history of smoking, with a higher proportion of males being smokers (48.1%) compared to females (5.6%, p=0.002, Table 1).

**Table 1:** Demographic and clinical characteristics CABG - coronary artery bypass grafting

Characteristic	Overall N=45	Male N=27	Female N=18	p-value
Age in years	29.42 ± 10.52	27.44 ± 10.30	32.39 ± 10.43	0.044
Age category				0.011
18 to <25	21 (46.7%)	17 (63.0%)	4 (22.2%)	
25 to < 30	11 (24.4%)	3 (11.1%)	8 (44.4%)	
30 or more	13 (28.9%)	7 (25.9%)	6 (33.3%)	
Weight (kg)	76.20 ± 18.84	76.63 ± 17.22	75.56 ± 21.55	0.880
Height (cm)	166.10 ± 7.86	170.20 ± 5.39	159.94 ± 6.96	<0.001
BMI (kg/m²)	27.57 ± 6.50	26.38 ± 5.43	29.36 ± 7.66	0.201
BMI category				0.118
Underweight	2 (4.4%)	1 (3.7%)	1 (5.6%)	
Healthy	16 (35.6%)	10 (37.0%)	6 (33.3%)	
Overweight	12 (26.7%)	10 (37.0%)	2 (11.1%)	
Obese	15 (33.3%)	6 (22.2%)	9 (50.0%)	
Diabetes	2 (4.4%)	1 (3.7%)	1 (5.6%)	>0.999
Hypertension	3 (6.7%)	0 (0.0%)	3 (16.7%)	0.058
Stroke	0 (0.0%)	0 (0.0%)	0 (0.0%)	>0.999
Myocardial infarction	0 (0.0%)	0 (0.0%)	0 (0.0%)	>0.999
Peripheral vascular disease	0 (0.0%)	0 (0.0%)	0 (0.0%)	>0.999
End-stage renal disease	0 (0.0%)	0 (0.0%)	0 (0.0%)	>0.999
History of CABG	0 (0.0%)	0 (0.0%)	0 (0.0%)	>0.999
Smoking	14 (31.1%)	13 (48.1%)	1 (5.6%)	0.002
Dominant hand				>0.999
Right-handed	42 (93.3%)	25 (92.6%)	17 (94.4%)	
Left-handed	3 (6.7%)	2 (7.4%)	1 (5.6%)	

Characteristics of the radial and ulnar arteries

**Figure 1:**
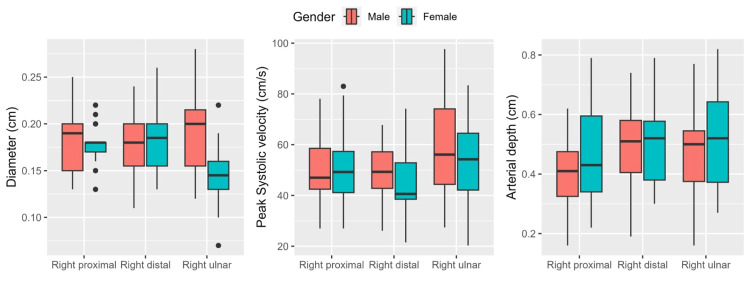
Gender-based differences in arterial parameters, including the diameter, peak systolic velocity, and depth

When comparing arterial characteristics between male (N=27) and female (N=18) participants within the cohort (N=45), statistically significant differences were observed in the diameter of the right ulnar artery (p=0.002). Specifically, in males, the mean diameter of the right ulnar artery was 0.19 ± 0.04 cm, while in females, it measured 0.14 ± 0.03 cm (Figure 1 and Table 2). No other significant differences in arterial diameter were noted between the genders. The peak systolic velocity and depth measurements for the various arteries did not demonstrate statistically significant differences between males and females (p>0.05).

**Table 2:** Characteristics of the radial and ulnar arteries across males and females

Characteristic	Overall N=45	Male N=27	Female N=18	p-value
Diameter (cm)				
Right proximal radial	0.18 ± 0.03	0.18 ± 0.03	0.18 ± 0.02	0.484
Right distal radial	0.18 ± 0.03	0.18 ± 0.03	0.18 ± 0.03	0.77
Right ulnar	0.17 ± 0.05	0.19 ± 0.04	0.14 ± 0.03	0.002
Peak systolic velocity (cm/s)				
Right proximal radial	51.04 ± 14.06	50.75 ± 13.33	51.48 ± 15.48	0.917
Right distal radial	48.07 ± 11.95	49.69 ± 10.68	45.64 ± 13.60	0.247
Right ulnar	57.70 ± 18.92	60.33 ± 18.72	53.77 ± 19.08	0.372
Right ulnar (with radial compression)	73.74 ± 22.29	76.93 ± 22.66	68.97 ± 21.47	0.271
Depth (cm)				
Right proximal radial	0.43 ± 0.13	0.41 ± 0.11	0.46 ± 0.16	0.307
Right distal radial	0.49 ± 0.13	0.49 ± 0.13	0.50 ± 0.14	0.945
Right ulnar	0.48 ± 0.15	0.46 ± 0.13	0.50 ± 0.17	0.385

Significant differences in arterial characteristics across BMI categories were observed in several parameters. Peak systolic velocity in the right PRA varied significantly across BMI categories (p=0.026), with values ranging from 33.55 ± 9.26 cm/s in the underweight category to 57.77 ± 12.47 cm/s in the obese category. Similar significant differences were noted in peak systolic velocity for the right DRA (p=0.013), right ulnar artery (p=0.006), and right ulnar artery with radial compression (p<0.001). Additionally, depth measurements in the right PRA (p=0.003), right DRA (p=0.007), and right ulnar artery (p<0.001) exhibited significant variability across BMI categories. However, no significant differences were found in arterial diameter across BMI categories (p>0.05, Table 3).

**Table 3:** Characteristics of the radial and ulnar arteries across BMI categories

Characteristic	BMI category				p-value
	Underweight N=2	Healthy N=16	Overweight N=12	Obese N=15	
Diameter (cm)					
Right proximal radial	0.19 ± 0.02	0.18 ± 0.03	0.18 ± 0.03	0.18 ± 0.03	0.989
Right distal radial	0.16 ± 0.04	0.18 ± 0.04	0.17 ± 0.04	0.18 ± 0.02	0.692
Right ulnar	0.12 ± 0.00	0.16 ± 0.04	0.19 ± 0.04	0.18 ± 0.05	0.078
Peak systolic velocity (cm/s)					
Right proximal radial	33.55 ± 9.26	47.36 ± 13.26	50.46 ± 14.42	57.77 ± 12.47	0.026
Right distal radial	32.25 ± 8.70	43.33 ± 11.20	48.38 ± 11.00	55.00 ± 10.06	0.013
Right ulnar	33.75 ± 8.98	48.28 ± 18.65	60.15 ± 11.12	68.99 ± 18.03	0.006
Right ulnar (with radial compression)	41.10 ± 15.70	60.39 ± 22.27	78.20 ± 15.89	88.77 ± 14.12	<0.001
Depth (cm)					
Right proximal radial	0.23 ± 0.10	0.37 ± 0.10	0.44 ± 0.11	0.51 ± 0.13	0.003
Right distal radial	0.31 ± 0.03	0.43 ± 0.11	0.54 ± 0.13	0.55 ± 0.12	0.007
Right ulnar	0.27 ± 0.15	0.39 ± 0.12	0.50 ± 0.15	0.58 ± 0.08	<0.001

Depth measurements in the right PRA displayed notable differences (p=0.013) among age groups, with values ranging from 0.39 ± 0.12 cm in the 18 to <25 age category to 0.52 ± 0.13 cm in the 30 or more age category. Similarly, depth measurements in the right ulnar artery exhibited significant variability (p=0.006) across age categories, with values ranging from 0.43 ± 0.13 cm in the 18 to <25 age category to 0.60 ± 0.12 cm in the 30 or more age category. However, no other significant differences were observed in arterial diameter or peak systolic velocity across age categories (p>0.05, Table 4). Of note, no statistically significant differences were found in arterial characteristics across smoking categories (p>0.05, Table 5).

**Table 4:** Characteristics of the radial and ulnar arteries across age categories

Characteristic	Age			p-value
	18 to <25 N=21	25 to <30 N=11	30 or more N=13	
Diameter (cm)				
Right proximal radial	0.18 ± 0.03	0.18 ± 0.02	0.18 ± 0.03	0.828
Right distal radial	0.17 ± 0.03	0.19 ± 0.03	0.19 ± 0.04	0.273
Right ulnar	0.17 ± 0.05	0.17 ± 0.05	0.17 ± 0.04	0.954
Peak systolic velocity (cm/s)				
Right proximal radial	49.76 ± 12.47	50.42 ± 15.98	53.64 ± 15.57	0.746
Right distal radial	47.88 ± 12.84	45.92 ± 14.38	50.21 ± 8.22	0.55
Right ulnar	54.15 ± 18.05	58.02 ± 24.43	63.18 ± 14.78	0.493
Right ulnar (with radial compression)	70.98 ± 23.44	67.82 ± 26.35	83.22 ± 13.60	0.121
Depth (cm)				
Right proximal radial	0.39 ± 0.12	0.40 ± 0.12	0.52 ± 0.13	0.013
Right distal radial	0.47 ± 0.12	0.51 ± 0.14	0.51 ± 0.15	0.418
Right ulnar	0.43 ± 0.13	0.44 ± 0.14	0.60 ± 0.12	0.006

**Table 5:** Characteristics of the radial and ulnar arteries across smoking categories

Characteristic	Smoker		p-value
	No N=31	Yes N=14	
Diameter (cm)			
Right proximal radial	0.17 ± 0.02	0.19 ± 0.03	0.066
Right distal radial	0.18 ± 0.03	0.19 ± 0.04	0.135
Right ulnar	0.17 ± 0.05	0.18 ± 0.04	0.273
Peak systolic velocity (cm/s)			
Right proximal radial	51.58 ± 13.91	49.86 ± 14.84	0.769
Right distal radial	47.17 ± 12.18	50.07 ± 11.62	0.364
Right ulnar	58.28 ± 19.43	56.43 ± 18.41	0.668
Right ulnar (with radial compression)	73.85 ± 21.78	73.51 ± 24.23	0.922
Depth (cm)			
Right proximal radial	0.43 ± 0.12	0.43 ± 0.16	0.731
Right distal radial	0.49 ± 0.13	0.50 ± 0.15	0.532
Right ulnar	0.47 ± 0.15	0.49 ± 0.15	0.668

**Figure 2:**
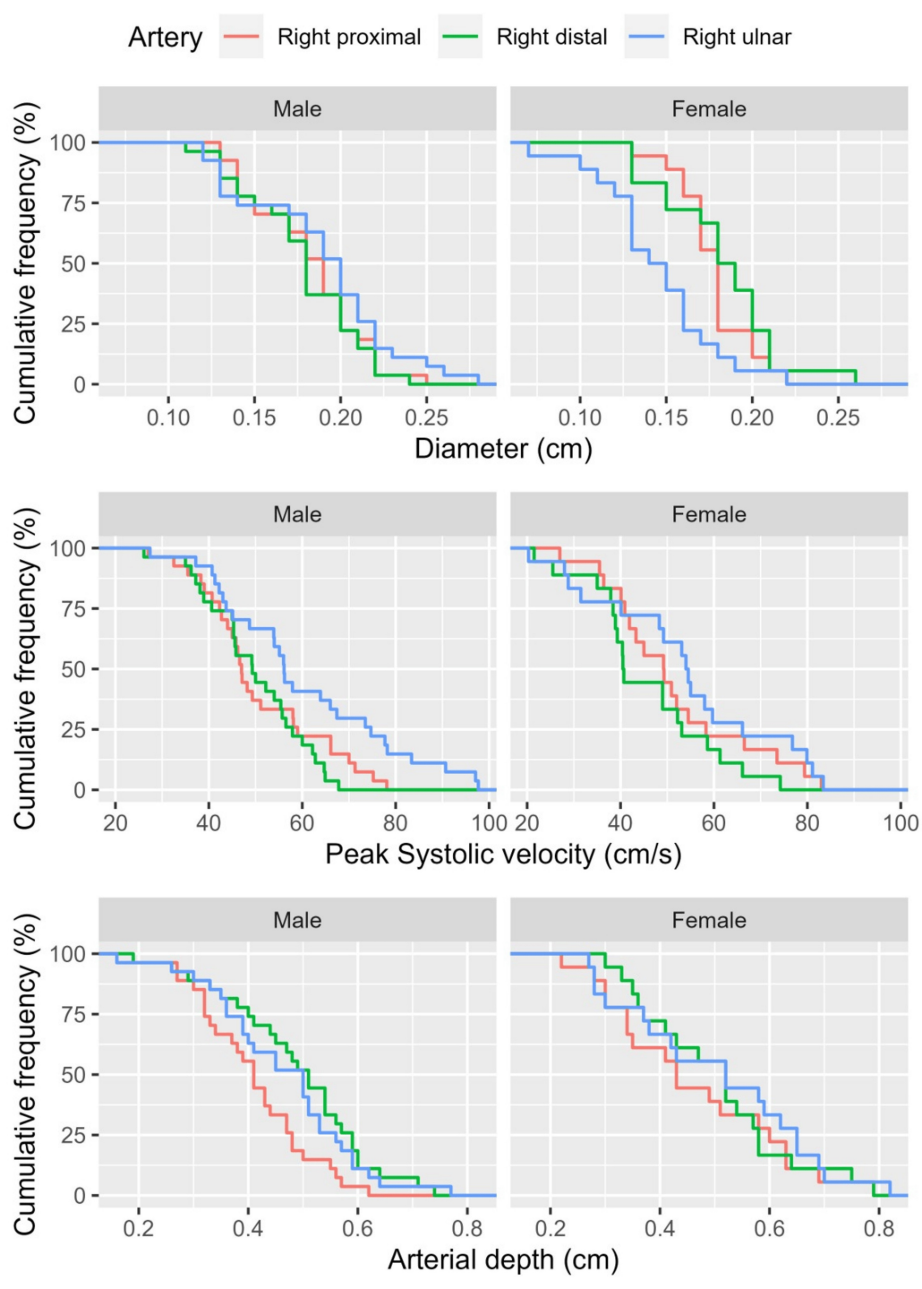
Cumulative frequencies of arterial parameters, including the diameter, peak systolic velocity, and depth among males and females under study

**Figure 3:**
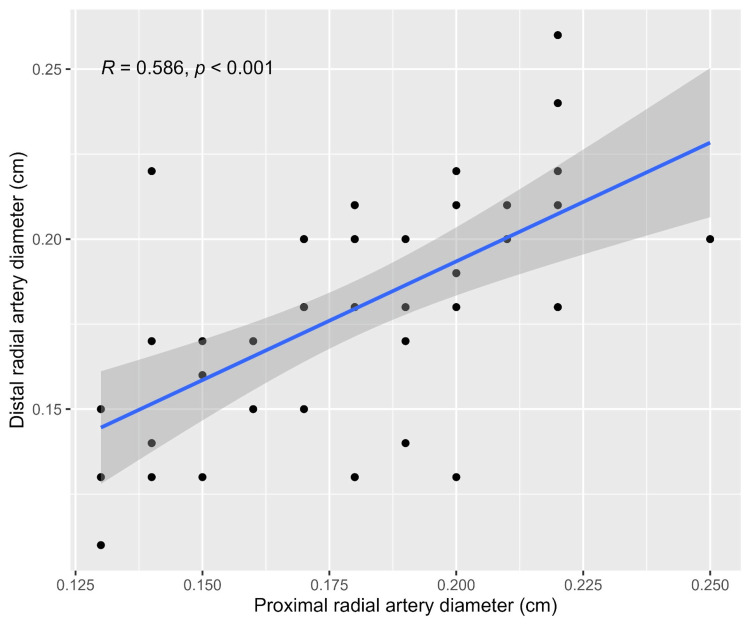
The correlation between the diameters of the proximal and distal arteries

A visual depiction of the cumulative frequencies of arterial parameters is demonstrated in Figure 2. Focusing on the bivariate correlation analysis, the results showed a positive correlation between the diameters of the PRA and DRA among patients (r=0.586, p<0.001, Figure 3).

Blood flow with radial compression in selected arteries

All the patients under study (100%) showed an intact wall of the right PRA, DRA, and ulnar arteries. The flow of the right thenar and dorsal digital arteries was normal in all patients (100%). However, two-thirds of patients had reversed flow with radial compression (66.7% in the right thenar and dorsal digital arteries), with no significant differences in the proportions of reversed flow among males and females (p=0.197 for both comparisons, Table 6).

**Table 6:** Statuses of the blood flow with radial compression in selected arteries

Characteristic	Overall N=45	Male N=27	Female N=18	p-value
Rt. Thenar artery				0.197
Not reversed	15 (33.3%)	11 (40.7%)	4 (22.2%)	
Reversed	30 (66.7%)	16 (59.3%)	14 (77.8%)	
Rt. Dorsal digital artery branches				0.197
Not reversed	15 (33.3%)	11 (40.7%)	4 (22.2%)	
Reversed	30 (66.7%)	16 (59.3%)	14 (77.8%)	

## Discussion

Our study identified several conclusions that are essential for understanding the properties of radial and ulnar arteries in Saudi Arabia. Several studies provided insights into the diameter of the DRA (Table 7). In our analysis of arterial characteristics between genders, as shown in Table 2, we found no significant difference in diameter measurements between males and females for both the right PRA and DRA. Both groups showed a mean diameter of 1.8 mm ± 0.3. In contrast to this finding, the Korean population exhibited a larger mean diameter of 2.31 mm, with a gender disparity of 2.43 mm in males and 2.15 mm in females [13]. Furthermore, data from the Sudanese population showed an even larger mean radial artery diameter of 2.5 mm, but without a significant difference between genders [17]. However, it is important to note that other studies have concluded that females have a significantly smaller DRA diameter than males, which may correlate with the higher success rates of DRA access procedures observed in men compared to women [18].

**Table 7:** Distal radial artery diameter comparison among studies

Criteria	Saudi study (present study)	Sudanese study (Taha et al., 2022) [2]	Korean study (Lee et al., 2022) [1]
Country	Saudi Arabia	Sudan	Korea
Method	Ultrasonography	Ultrasonography	Ultrasonography
No. of patients	45	28	1162
Male	27	14	671
Female	18	14	491
Diameter of the right distal radial artery			
Total	0.18 ± 0.03 cm	0.25 cm	0.232 ± 0.044 cm
Male	0.18 ± 0.03 cm	0.25 cm	0.243 ± 0.044 cm
Female	0.18 ± 0.03 cm	0.25 cm	0.216 ± 0.038 cm

As for the ulnar artery, our results show a significant difference in the diameter of the right ulnar artery between males and females. Males showed a larger mean diameter of 1.9 mm compared to 1.4 mm for females, while individuals from Sudan maintained more uniform diameters of 2.2 mm across both genders [17]. The variation in arterial diameters, as shown by the comparison with Korean and Sudanese data, suggests that both ethnic background and gender play significant roles in arterial dimensions.

No statistically significant differences were found between males and females in peak systolic velocity and depth measurements for any of the measured arteries (p>0.05). This suggests that even though the diameter of the right ulnar artery varies across genders, the blood flow velocity and depth of the arteries remain the same.

Table 3 shows significant variations in arterial characteristics that correlate with BMI categories. The peak systolic velocity in the right PRA significantly differs across BMI categories (p=0.026), ranging from 33.55 ± 9.26 cm/s in underweight individuals to 57.77 ± 12.47 cm/s in obese individuals. This suggests that an individual's BMI may have an effect on the velocity at which blood flows through a radial artery. In contrast, the diameter of the right PRA and DRA does not show a significant change with varying BMI, indicating that vessel size may be less impacted by BMI than blood flow velocity.

Regarding arterial depth, there is a distinct pattern. The depth of the right PRA increases from 0.23 cm in underweight individuals to 0.51 cm in obese individuals, which represents a statistically significant change with a p-value of 0.003. The right DRA depth follows a similar pattern, ranging from 0.31 cm in underweight individuals to 0.55 cm in obese individuals, as confirmed by a p-value of 0.007. Additionally, the depth of the right ulnar artery increases from 0.27 cm to 0.58 cm across the same BMI spectrum, with a highly significant p-value of less than 0.001. These findings highlight the correlation between increased BMI and greater arterial depth, which may have clinical implications, particularly in arterial access procedures.

Analyzing arterial characteristics across different age categories, as shown in Table 4, yields mixed results. Arterial diameter does not show a significant difference with age, as indicated by p-values of 0.828 for the right ulnar artery, 0.273 for the right DRA, and 0.954 for the right PRA. This indicates that the size of these arteries does not change markedly with age among the studied groups. Interestingly, this contrasts with findings from an external study, which indicated that vessel diameter tends to become smaller with advanced age [17].

Furthermore, we observed that peak systolic velocities within the right PRA and DRA, as well as the right ulnar artery, do not show significant variations across the age groups (p-values of 0.746, 0.550, and 0.493, respectively), indicating that the functional blood flow characteristics are maintained with aging. However, our data shows a significant age-related increase in the depth of the right ulnar artery. The depth measurements increased from 0.43 ± 0.13 cm in the youngest age group (18 to <25) to 0.60 ± 0.12 cm in the oldest group (30 or more), as indicated by a p-value of 0.006. This suggests that with advancing age, the ulnar artery tends to be located deeper within the arm tissues, which may have clinical implications.

Table 5 shows a comparison of the characteristics of the radial and ulnar arteries between smokers and non-smokers. The impact of smoking on arterial characteristics did not show significant differences across the measured parameters. Diameters, depth, and peak systolic velocities of the right PRA, right DRA, and right ulnar arteries did not exhibit notable differences between smokers and non-smokers, with p-values significantly exceeding the threshold. However, this contrasts with other research that has demonstrated a reduction in arterial diameter associated with smoking [19].

Considering all the results, this study's findings have several implications for future research and practice. The observed variations in arterial characteristics based on factors such as age, BMI, and gender may impact the management and treatment of arterial diseases. As it stands, this could also influence the approach to any condition related to arteries. Likewise, these characteristics could be further examined in future studies to identify potential factors contributing to gender and age differences. Moreover, further research should investigate the significance of reversed flows with radial compression in the right dorsal and thenar digital arteries, as well as the implications of the positive correlation between PRA and DRA diameters.

Study limitations

The study has several limitations that need to be acknowledged. Firstly, the small sample size from a single area prevents an accurate reflection of the general population. Additionally, possible biases in the volunteer selection process are another limitation. To corroborate these findings, further research with larger and more diverse populations would be beneficial.

## Conclusions

In conclusion, our research on the ultrasonographic characteristics of the radial artery in Saudi Arabians may offer valuable clinical correlations. While factors such as gender may not significantly impact arterial diameter, age and BMI, including peak systolic velocity and depth, do. It is worth noting that Saudi Arabian arteries have catheters of different sizes compared to those of Korean and Sudanese individuals. Further studies should be conducted to validate these findings or explore their demographic determinants. Our report suggests that ultrasonography demonstrates comparable levels of accuracy in identifying the appropriateness of the radial artery for treatment.
